# Notes and News

**Published:** 1888-11-24

**Authors:** 


### NOTES AND NEWS.

Collected and Communicated.

An account is given in a recent number of the *Gazetta Musicale* of Milan, of a hospital at Villanova built and supported by Verdi, the composer. The hospital is close to Verdi's private residence, and was opened on November 6th, in the simple manner of throwing wide the doors and taking in twelve sick persons who applied for relief.

The committee of the Stockton Surgical Hospital have sanctioned the erection of a new wing at a cost of £2,000. Towards this they have £1,000 invested. The new wing is to hold thirty beds. Until this necessary addition can be opened the Preston Female Ward is to be given up to men, and no more women patients will be received except the victims of accidents.

The secretary of the National Dental Hospital, Great Portland Street, announces that in future ladies can be trained as dentists at the hospital. Last year the doors were thrown open to women medical practitioners, and the special advantage of admitting those who hold no diploma, is that Zenana missionaries will be able to secure dental instruction denied them elsewhere.

The net total of the receipts of Madame Patti's four charity concerts at Swansea in 1882, 1884, 1886, and the present year was £3,106, of which £2,113 were given to Swansea Hospital, and the remaining £993 were divided among the poor of the Craig-y-nos district of the Swansea Valley. There are not many people who could raise £3,000 pounds by four charity concerts.

The Committee of the Warneford Hospital, Leamington, have decided to buy a vacant strip of land to the east of the hospital, belonging to Lord Aylesford. The hospital is constantly overcrowded, and as soon as funds permit, further building operations must be commenced. An anonymous friend has given £500 towards the purchase of this only piece of available ground, and Mrs. Hitchman has given £200. The total required is £1,850.

The Nursing Staff of the Hull Royal Infirmary have presented a handsome clock in a carved oak case, with a suitable inscription, to Mr. Edward Harrison, F.R.C.S., on the occasion of his leaving the infirmary, after having held the post of house surgeon for three and a-half years. Mr. Harrison's services have been greatly appreciated, and his future career will be watched with interest by many who have learnt to value his kindness and skill during his useful work at Hull.

At the late meeting of the Woolwich and Plumstead Hospital Committee, on the motion of Mr. Rabson, the following resolution was carried: "That a contract be prepared to expend £1,500 towards the erection of this hospital, in accordance with the plans and specifications approved by the War Office, and that a further contract be entered for the completion of the building when funds permit." The Committee have £1,500 in hand, and will doubtless accept the tender of Mr. Coombes for building the hospital.

The Female Orphan Asylum at Beddington, Surrey, has sheltered nearly 4,000 orphans since it was founded in 1758. The girls are trained especially for domestic service, and when they are fit are found places in respectable Protestant families. If the girls do well the committee grant them small gifts in money at intervals. Owing to extra expenses connected with drainage, the committee have been obliged to sell some of their stock. The expenditure of the asylum largely exceeds the income.

The North Staffordshire Infirmary has an Incurable Fund, which during the last year has benefited nine recipients. This seems a good idea for a county infirmary. Altogether, the working of this Infirmary seems satisfactory, for with 581 operations, including abdominal sections, there have only been four deaths. The Hospital Sunday collections amount to £300 more than last year, and the musical festival produced a sum of £200, so that the funds of the Infirmary are in a fairly satisfactory state.

When Mr. Gladstone was departing from Birmingham after the great meeting there, he presented Lady Foster with a cheque for the purchase of toys for the Children's Hospital. Lady Foster put the cheque in her purse, and her purse in her pocket, and the whole proceeding was doubtless watched by some clever thief. In the crush and excitement which occurred at the starting of the train, Lady Foster's pocket was picked, and the cheque meant for the children fell into alien hands.

The annual meeting of the governors of the Isle of Wight Infirmary took place on November 9th; 468 in-patients, 2,081 out-patients, 683 dental patients, and 103 in the convalescent home, gave a total of over 3,000 persons benefited during the year. The new wing, containing an operating-room, sitting-room, and three bed-rooms for nurses, is furnished, but not occupied yet. The position of this charity has greatly improved during the last year, in spite of an annual loss of £74, owing to the fall in Consols. It is proposed by Dr. Davey to start lectures on nursing, and to try and make the hospital a training school for nurses.

A letter published in the *Edinburgh Scottish Leader* accuses the infirmary authorities of inattention. The writer says: "Late on Saturday night, between eleven and twelve I think, a young man accidentally got two of his fingers jammed by a door, and very much injured. He went straight to the infirmary to have them dressed, but whether it was too much trouble to do it then or not I cannot say, but, at all events, after waiting two hours, he was asked to come back at eleven o'clock in the forenoon; he did so, and then had to wait three hours before he was attended to." Doubtless upon inquiry this will prove to be another "case against a hospital," such as are always being disproved and corrected.

The invalid children of London have got an association all of their own, which ought to be able to brighten many dreary little lives. To be ill in a crowded room, and to be ill for long years, or perhaps for ever, is an existence the trials and sufferings of which, but few of us can imagine. The Charity Organisation Society has had a happy thought, and hopes to attach to every poor crippled or bedfast child in London, a lady visitor, who will do her best to introduce a little cheerfulness and variety into the life of her special protegé. The object is not so much to supply strengthening food, or nursing appliances, as to provide a friend who will be patient with the poor child, and converse with him or her on pleasant subjects, and perhaps lend books to wile away the weary hours. It is a very simple scheme, and since the Society has every means through its officers of learning all about the little invalids, it only remains for visitors to be found, and success is assured. Contributions and offers of help should be sent to the office, 18, Buckingham Street, Charing Cross.

THE following vacancies are announced this week, the amount of salary in each case being given after the name of the institution :—

**Resident Medical Officers.**

Stroud General Hospital. House Surgeon.—£80, board, etc.
Blackburn Infirmary. Junior House Surgeon.—£30, board, etc.
Bradford Infirmary and Dispensary. House Physician.—£100, board, etc.
Kent and Canterbury Hospital. Assistant House Surgeon and Dispenser.—£50, board, etc.
Manchester Friendly Societies' Medical Association. Surgeon.—£150, house, etc.
Royal Hants County Hospital. House Surgeon.—£100, board, etc.
Free Hospital for Children of the Poor, Kingsholm. Surgeon.
Oxford University. Deputy Professor of Human and Comparative Anatomy.—£500.

**Non-resident Medical Officers.**

Victoria University. Joint Lecturer on Forensic Medicine.
Royal South Hants Infirmary. Assistant Surgeon.
Hospital for Epilepsy, Portland Terrace. Physician to Out-Patients.
Hospital for Sick Children, Great Ormond Street. Assistant Surgeon.
Kensington Dispensary. Honorary Surgeon
Lock Hospital, Soho. Surgeon to Out-Patients.
National Dental Hospital, Great Portland Street. Lecturer on Dental Anatomy and Physiology.
Royal South London Dispensary. Surgeon.—£20.

ON the vote of £32,802 for the Broadmoor Criminal Lunatic Asylum coming before the Parliamentary Committee, Mr. Labouchere took the opportunity of making strictures on some of the asylum expenses. He said the asylum was a nest of jobbery; there were 151 officials to look after 541 lunatics. The chaplain received £400 from the asylum, and £390 as a retired instructor in the navy; in addition, there was £18 charged for substitutes in the chaplain's absence. Nobody paid much attention to Mr. Labouchere's remarks, but Dr. Farquharson took the opportunity of bewailing the neglect of the teaching material offered by this large asylum.

SIR MORELL MACKENZIE delivered the inaugural address at the first meeting of the British Association of Laryngologists. Taking the subject made familiar in Lionel S. Beale's book on "Slight Ailments," Sir Morell dwelt on the importance of giving attention to minor illnesses, and remarked that these were often neglected by doctors, because such cases were not "interesting." If medical men would show courageous humility by giving their best abilities to the simplest cases, the patients would be very often much more rapidly cured. He also spoke of the importance of always endeavouring to cure cases, however hopeless they appeared, observing that there were many cases which medical men did not try to cure because they did not hope to cure them.

AT the opening of the meeting of the Royal Colonial Institute for this session, Dr. Symes Thompson read an interesting paper on "South Africa as a Health Resort." The facts given were gathered during a recent tour undertaken by Dr. Symes Thompson to the Cape. A vast change (the lecturer said) had come over the world as regards holidays, and the development of the railway system had brought within reach many places previously inaccessible. He could recommend a a short pleasure trip to South Africa to those seeking new grounds for their annual holiday just as much as to invalids. When in Cape Town he had exceptional opportunities of discussing questions connected with the climate. The chief characteristics of the air were dryness and clearness. The seasons were regular; from June to October no rain, but from November to May an abundant rainfall. At an altitude of 4,000 ft. or 5,000 ft. the climate had been compared to a Devonshire summer. In conclusion, he trusted that, though the time had not come for a consolidation of the various colonies, the solidarity of South Africa would one day be accomplished by pacific means. A discussion followed, in which Sir Charles Mitchell, Dr. Lennox Browne, and others took part.

IT seems that a bazaar may sometimes result in danger to the body as well as the purse. At the Cambridge Police Court last week Humphry Lloyd Bradshaw, undergraduate of Christ's College, was fined £5 for assaulting Mr. Rowton, a governor of Addenbrooke's Hospital. The defendant and friends, who had been dining, went to a bazaar which was being held on behalf of the hospital, and the defendant bonneted the complainant. Mr. Rowton, however, had his wits about him, and promptly gave the undergraduate in charge. There was an attempted rescue, but without result. Humphry Lloyd Bradshaw would have done better to have spent that £5 at the bazaar.

A WRITER in the *Englishwoman's Review* is astonished that all the daily papers should have quoted such a stupid article as that of THE HOSPITAL on "Women and their Victims." An accusation of cruelty to animals preferrred by men against women, says this indignant writer, is ludicrously absurd; for it is man's mission to destroy life, and woman's mission to preserve it. If the truth were known women only wear bird's wings, &c., because the men shoot so many harmless little dicky-birds, that it is a pity not to get some economy out of a cruel amusement. We wish this writer had addressed her diatribe to our office, we would gladly have printed her letter for the amusement of our readers.

MR. E. ST. AUBYN made a capital speech at the annual meeting of the governors of the Royal Albert Hospital, Devonport, which was held last week. This hospital has been improving rapidly lately: they have secured teak floors for all the wards; they have improved the drainage, have started a nursing institute, and have established an ophthalmic department, which has had 400 patients. The lock wards, started at the request of the Government, are working well, and the matron and chaplain take great trouble to procure homes for those discharged cured from these wards. To help this latter work an appeal was issued, the response to which was so little as not to cover the expenses of printing and postage. Yet to cure these patients, and then to discharge them to their former life, is a very poor sort of charity.

COUNCILLOR D. SHARROCKS has addressed a letter to the Salford Town Council about the proposed infectious hospital. He contends that it is the duty of the Council to provide adequate isolation and efficient accommodation for cases of infectious disease, but at the same time to do so on business and common-sense principles, not with extravagant ideas. Mr. Sharrocks tells the story of Monsall Hospital as an example of how economically and thoroughly a fever hospital can be managed, and he compares the expenditure at Monsall with the expenditure at Wilton, which has cost Salford a loss, estimated by Mr. Sharrocks, at £25,000. The following figures relating to the two hospitals in question are quoted :—

HOUSE EXPENSES.—MEAT, GROCERIES.

| | | | |
|---|---|---|---|
| Monsall, | 1876-86, | 12,403 cases. | Cost per case, £1 16s. 6d. |
| Wilton, | 1876-86, | 3,877 cases. | Cost per case, £2 8s. 5d. |

SALARIES AND WAGES.

| | |
|---|---|
| Monsall | £1 5s. 7½d. per case. |
| Wilton | £2 1s. 5d. per case. |

MEDICINE AND STIMULANTS.

| | |
|---|---|
| Monsall | 3s. 6d. per case. |
| Wilton | 17s. 1d. per case. |

Wilton Hospital does not give wines and spirits as a separate item, as Monsall does; but the elective auditor has supplied the information, and it shows as follows :—

| | |
|---|---|
| Monsall | 0s. 11¾d. per case for 12 years. |
| Wilton | 6s. 7d. for last year. |

In conclusion, Mr. Sharrocks strongly recommends the Salford Council to come to some arrangement with the Monsall authorities, and not to persist in building what he is convinced will be an extravagant and costly blunder.

